# Yearlong Near-Syncope: Presentation of a Rare Right Atrial Myxoma

**DOI:** 10.7759/cureus.59070

**Published:** 2024-04-26

**Authors:** Vishal V Bandaru, Sophie L Talbot, Ricardo I Garcia, Luis J Carbajal, Pooja Sethi

**Affiliations:** 1 Cardiology, Texas Tech University Health Sciences Center, Lubbock, USA

**Keywords:** heart surgery, cardiology, benign cardiac tumor, right atrial myxoma, near syncope

## Abstract

Atrial myxomas are the most common form of primary benign cardiac tumors. The left atrium is typically the most common location while right atrial myxomas are much rarer and only occur in about 15%-25% of all myxoma patients. Typically, left atrial myxomas have the ability to cause symptoms such as syncope. We report a case of a 67-year-old female who presented with complaints of palpitations, dizziness, and near-syncope that had been ongoing for about a year. Other causes of syncope were investigated and ruled out. A transthoracic echocardiogram (TTE) found a large 4.3 x 4.0 cm spherical mass in the right atrium which was confirmed by surgical resection and immunohistochemistry to be a myxoma. The patient's condition of syncope-like symptoms warrants elevating atrial myxomas to a higher position in the diagnostic differential.

## Introduction

Cardiac tumors are rare; the incidence of tumors found on autopsies ranges from 0.001% to 0.3% and are reported to be 0.2% of all tumors found in humans. Roughly 75% of cardiac tumors are benign. Approximately 50% of these benign tumors are myxomas [1-5]. Characteristic findings of atrial myxomas include syncope, dizziness, orthopnea, pulmonary edema, cough, palpitations, fatigue, hemoptysis, and chest pain. Many of these findings are influenced by changing positions. Right atrial myxomas have been found to be associated with abdominal distension due to ascites [2,4]. Another study evaluating atrial myxomas found a neurological symptom rate of 16.9% with cerebral infarction being the most common complication among neurological conditions [6]. Auscultation can reveal a “tumor plop” heart murmur [4]. Diagnostically, atrial myxomas have generally been found via 2D-Echocardiograms such as a transthoracic echocardiogram (TTE), transesophageal echocardiogram (TEE), or via computerized tomography (CT) or magnetic resonance imaging (MRI) [7].

## Case presentation

The patient is a 67-year-old female with a past medical history of hypertension (HTN), hyperlipidemia (HLD), type 2 diabetes (DM2), and Parkinson’s Disease who was admitted to the hospital in December 2022 for evaluation of palpitations associated with dizziness and near-syncope ongoing for one year. Episodes at onset lasted a few minutes before spontaneously resolving but worsened over the year in frequency and duration. She was referred to an outpatient cardiology clinic in December 2022 where a transthoracic echocardiogram (TTE) showed a right atrial mass concerning myxoma. The patient was sent to the emergency center for further evaluation and subsequently admitted for cardiovascular surgery evaluation. The physical examination was negative for murmurs, rubs, gallops, or clicks. An electrocardiogram (ECG) was unremarkable, and vitals were stable. Lab workup showed mildly elevated pro-brain natriuretic peptide (proBNP) of 259 pg/mL but otherwise was unremarkable.

**Figure 1:**
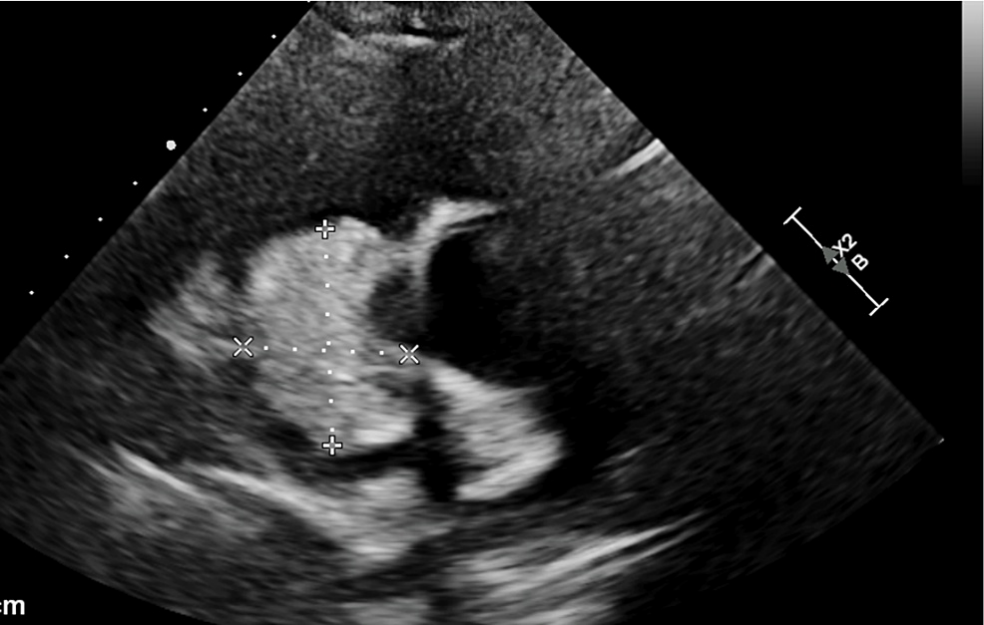
TTE parasternal short axis view at the level of the aortic valve depicting a large 4.3 x 4.0 cm spherical mass arising in the right atrium TTE: transthoracic echocardiogram

**Figure 2:**
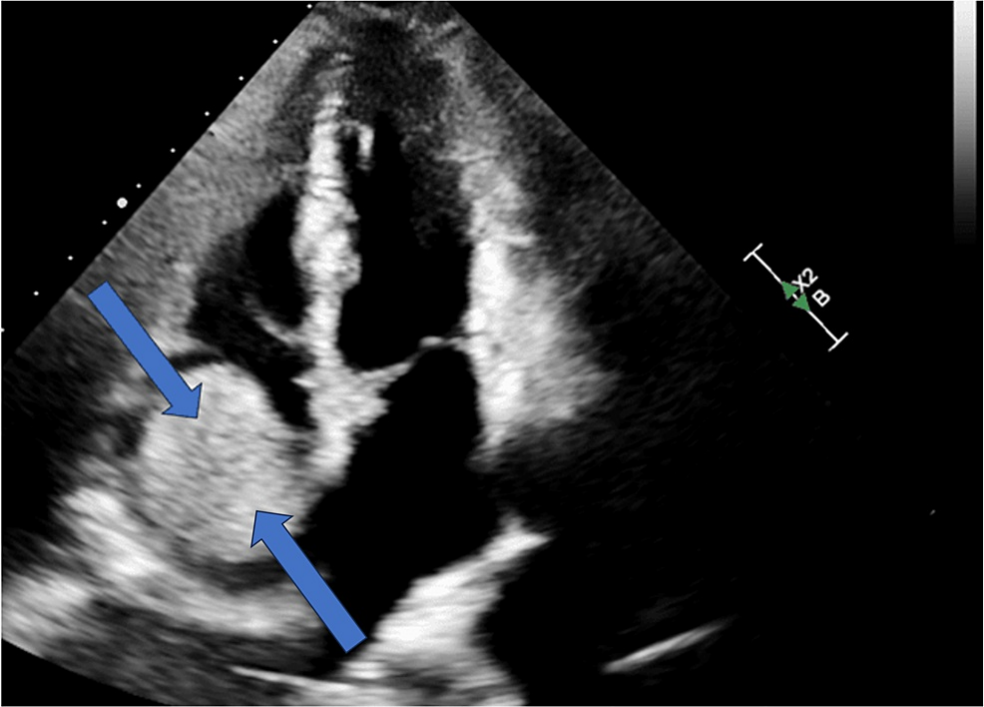
Apical four-chamber view of TTE again depicting a large mass in the right atrium (blue arrows) TTE: transthoracic echocardiogram

**Figure 3:**
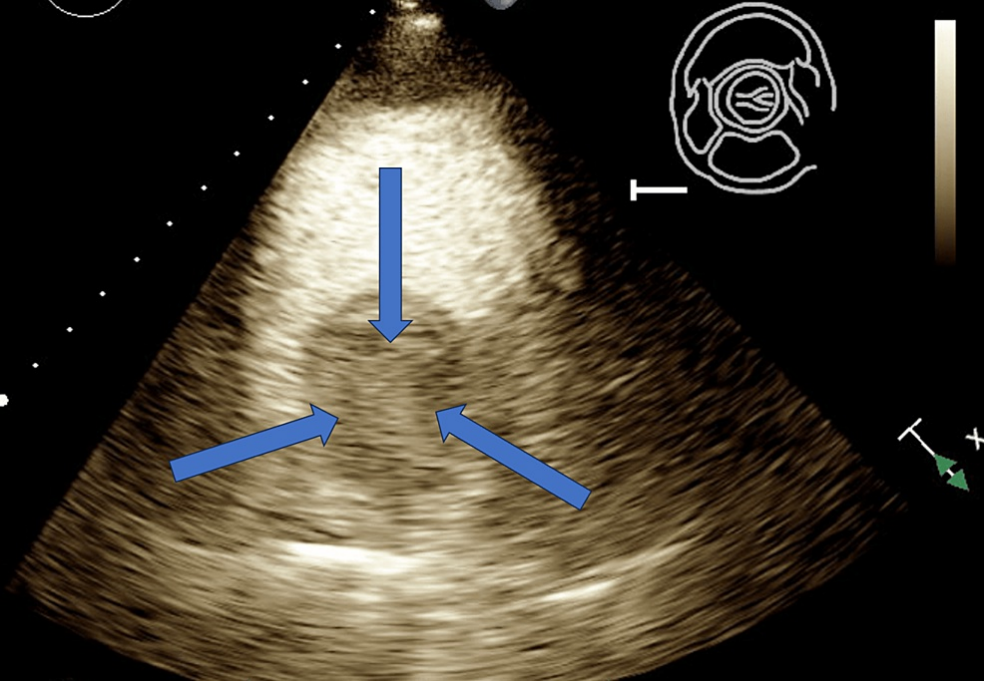
TTE parasternal short axis view at the level of the aortic valve with IV contrast depicting partially vascularized right atrial mass concerning myxoma (blue arrows) TTE: transthoracic echocardiogram

A repeat TTE was done after admission which revealed a large 4.3 x 4.0 cm spherical mass arising in the right atrium and a small pericardial effusion without tamponade physiology. This mass appeared at least partially vascularized in echo contrast, raising suspicion of a myxoma (Figures 1-3).

**Figure 4:**
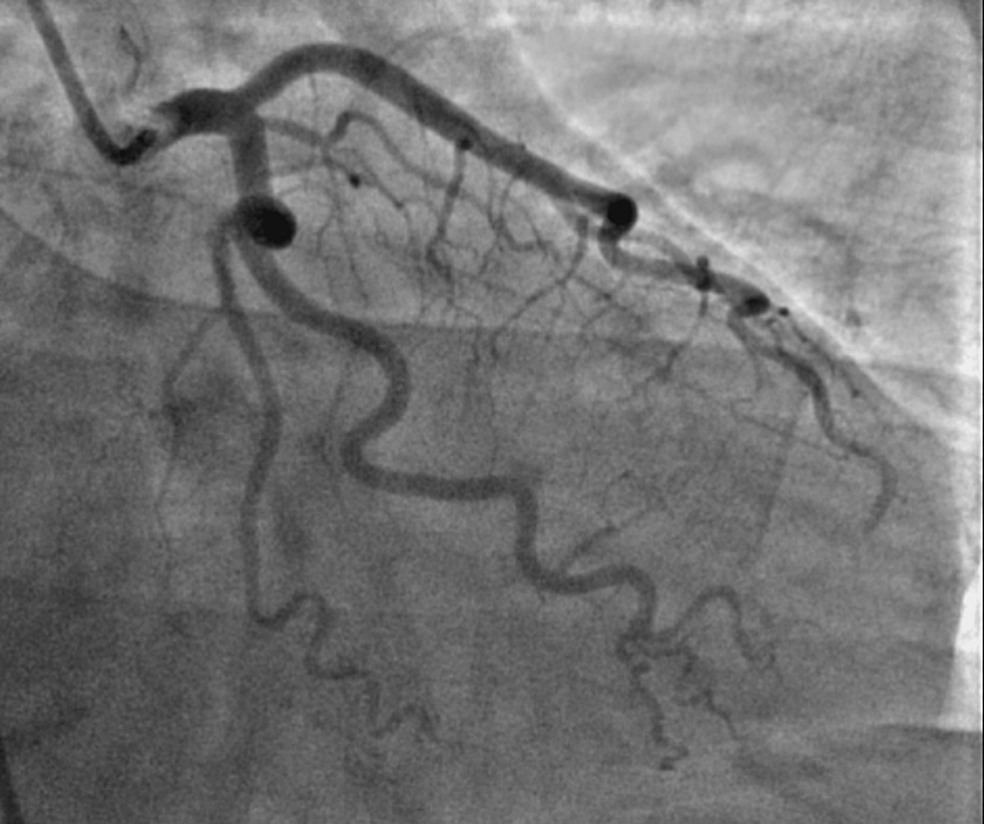
Right anterior oblique (RAO) view of left heart catheterization (LHC) with no significant left coronary artery disease

**Figure 5:**
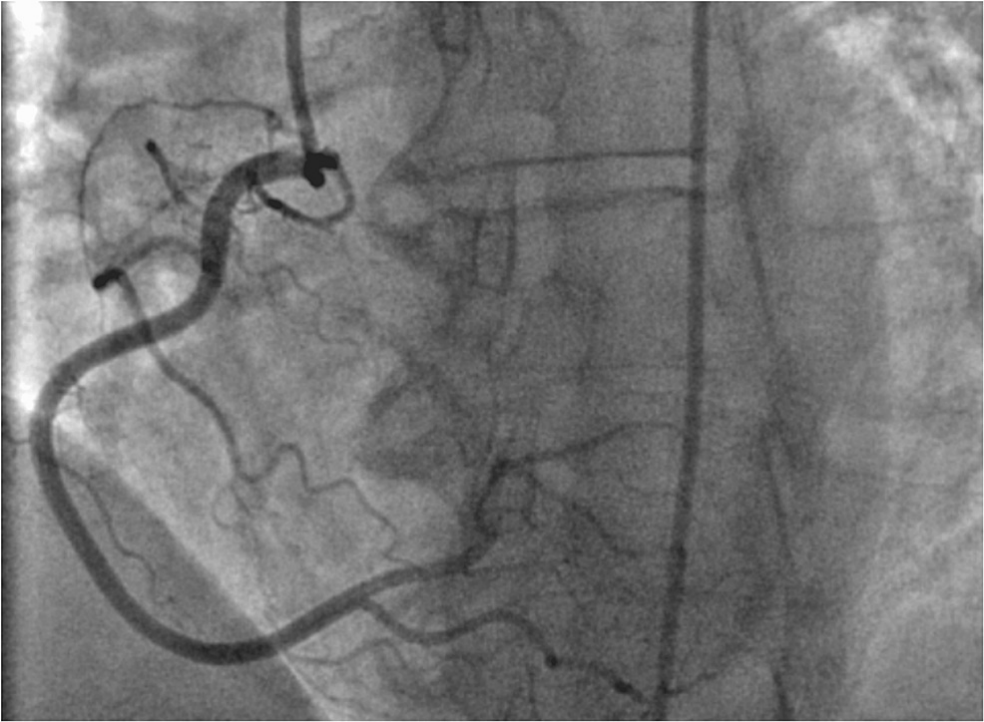
Left anterior oblique (LAO) view of the left heart catheterization (LHC) with no significant right coronary artery disease

Tissue Doppler imaging showed a mitral inflow pattern consistent with impaired relaxation of the left ventricle associated with grade I (mild) diastolic dysfunction. Left heart catheterization (LHC) was performed which showed no significant coronary artery disease (Figures 4, 5). There were normal cardiac filling pressures, normal ejection fraction (EF) without regional wall motion abnormalities, and normal coronary arteries.

Our patient was placed on cardiopulmonary bypass and underwent sternotomy with successful resection of the right atrial singular circular mass. No other evidence of masses was found. No blood was given intra-operatively or post-operatively due to the patient’s status as a Jehovah’s Witness. Frozen sections of the excised mass were sent to pathology; three different specimens were selected and the sections showed polygonal/stellate myxoma (lepidic) cells around blood vessels within the myxoid stroma, positive for Calretinin stain, consistent with cardiac myxoma. In additional stains, S100 was negative and CD 68 highlighted scattered histiocytes. The immunohistochemistry (IHC) supported the diagnosis of an atrial myxoma. There were no post-operative complications and the patient was discharged home in stable condition after three days.

## Discussion

Syncope is considered a rare manifestation of right atrial myxomas, seen in approximately 20% of cases [8-10]. Many case studies have found right atrial myxomas with a history of syncope and/or heart palpitations [8-13]. However, the absence of thromboembolic causes of syncope or common clinical findings suggestive of alternate etiologies of syncope should allow myxomas to be included higher in the differential, particularly in older patients [8]. Near-syncope symptomatology, over a long period of time, may need to garner further attention to right atrial myxomas as a possible cause. While syncope could be associated with reflex syncope, orthostatic hypotension, drug interactions, or dehydration, if the evaluation of the patient is still not explained or has additional cardiac symptomatology, a TTE may be a viable and easy mechanism for the diagnosis of myxomas [14]. 

Between 75-80% of myxomas are found in the left atrium and 15-20%, are located in the right atrium. Though myxomas may appear in other areas such as ventricular, biatrial, and multilocular locations, these myxomas represent an incredibly small proportion of cases [15]. Atrial myxomas have been found more often in women and over the atrial septum [4]. Generally, left atrial myxomas can cause syncope due to occlusion of the mitral valve. However, our case is most likely caused by a partial occlusion of the superior vena cava. Right atrial myxomas can cause symptoms resembling syncope if there is an obstruction to either the mitral valve or superior vena cava.

Our specific case had no irregularities in the myxoma in regards to the shape, size, and IHC. This myxoma was round, a shape that has been commonly reported in the literature: myxoma shapes have been recorded as round, multilobulated, or other irregular shapes. The size was within the reported literature value ranges (5.1 cm + 1.8 cm diameter) [16]. However, the presence of vascularization in the myxoma observed during contrast echo is an interesting finding. Due to the similarity in IHC between atrial myxomas and mural thrombi with myxoid stroma, the calretinin marker is specifically used for the differential, which was positive in our histochemistry. Other diagnostic considerations include intracardiac thrombi, rhabdomyomas, lipomas, sarcomas, or B-cell lymphomas [4,17]. Survival for patients with atrial myxomas is quite good at around 96% for a 10-year survival period and surgical interventions usually resolve symptoms [18].

## Conclusions

The prevalence of right atrial myxomas is considerably less than that of left atrial myxomas. Older, female patients are more likely to have atrial myxomas. Surgical resection is often the choice of treatment for suspected myxomas which has historically resolved symptoms. Excisional biopsy and IHC are often necessary to confirm the diagnosis of a myxoma and exclude other possibilities. Our patient's yearlong palpitations and near-syncope symptoms were finally resolved post-surgery, but the duration of the symptomatology begs the overall question of differentials. This symptomatology warrants a deeper investigation into placing myxomas higher in the diagnostic differential for older patients.
